# What Are the Positives? Exploring Positive Welfare Indicators in a Qualitative Interview Study with Livestock Farmers

**DOI:** 10.3390/ani9090694

**Published:** 2019-09-17

**Authors:** Belinda Vigors, Alistair Lawrence

**Affiliations:** 1 Scotland’s Rural College (SRUC), West Mains Road, Edinburgh EH9 3RG, UK; alistair.lawrence@sruc.ac.uk; 2 Roslin Institute, University of Edinburgh, Penicuik, EH25 9RG, UK

**Keywords:** positive animal welfare, positive animal welfare indicators, farmer attitudes, farmer knowledge, qualitative research, free elicitation narrative interviewing

## Abstract

**Simple Summary:**

Positive animal welfare is a relatively new concept which promotes the welfare benefits of providing animals with greater opportunities for positive experiences, in addition to minimising negative experiences. However, little is known about farmers’ attitudes towards this, and their knowledge of positive welfare. This presents a significant hurdle for the promotion of positive welfare indicators on-farm, where their effective implementation may depend on their acceptance by farmers. In response, this study uses qualitative interviews to explore farmers’ positive welfare perspectives. It finds that several aspects, reflective of the literature on positive welfare indicators, are evident in farmers’ positive welfare-related discussions. These include animal autonomy, play, positive affect, positive human–animal relationships, social interaction and appropriate genetic selection. Such findings provide insights into what farmers consider are relevant to, and indicative of, positive welfare. In addition, this paper explores how farmers see their role in the provision of positive welfare. It finds that farmers largely consider their inputs should be focused on making sure their animals’ needs (e.g., resources) are met and negative experiences are reduced, and that positive welfare will arise naturally, or indirectly, out of this. The implications of these findings and their overlaps with the positive welfare literature are discussed.

**Abstract:**

To support the furtherance of positive animal welfare, there is a need to develop meaningful and practical positive welfare indicators for on-farm welfare assessment. Considering the perspectives of farmers is arguably critical in this regard. Doing so helps ensure positive welfare indicators reflect farmers’ existing welfare norms and attitudes and, are thus, of practical relevance to them. However, a key issue for such development is the dearth of knowledge on farmers’ perspectives of positive welfare. To address this, this study uses qualitative interviews to directly examine livestock farmers’ perspectives of positive welfare. Findings reveal that farmers describe elements of positive welfare which are broadly in line with indicators suggested in the positive welfare literature. These elements include animal autonomy, play, positive affect, positive human-animal relationships, social interaction, and appropriate genetic selection. Additionally, this study finds that farmers construct the reduction of negative aspects of welfare as their primary management concern and mostly construct positive welfare as arising indirectly from this. Insights into the importance that farmers of different sectors and systems give to different aspects of positive welfare indicators are also explored. The implications of these findings and the similitudes between farmers’ perspectives and the positive welfare literature are discussed.

## 1. Introduction

There is no singular definition of positive animal welfare. However, one broad conception is that it emphasises the welfare relevance of providing animals with opportunities to have positive experiences, in addition to minimising negative experiences [1,2]. As positive animal welfare continues to develop, its furtherance requires suitable indicators for on-farm positive welfare assessment. Here, the primary concern of welfare science will be ensuring such indicators are reliable, valid and feasible [3]. However, research has found that farmers may mistrust the reliability of animal-based welfare indicators [4]. As a result, they may resist changing to systems which increase opportunities for animals to engage in natural behaviours because of economic viability concerns (e.g., cost of investment) [5,6]. This has implications for positive animal welfare, which predominantly focuses on animal-based indicators such as the presence or quality of play behaviours [3,7], social interaction [8] and curiosity driven exploration [9,10]. Engaging with farmers to understand their perspectives on positive welfare and considering this in the development of positive welfare indicators may be critical for their acceptance and implementation at farm level.

Research finds that farmers acceptance and/or implementation of welfare indicators can be influenced by how useful they perceive particular welfare indicators to be, based on their current welfare attitudes and values [4]. In addition, the ability of welfare indicators to function as a decision support tool and be adaptable to local farm conditions can further influence their perceived usefulness [11,12]. Thus, to develop positive welfare indicators which farmers perceive as useful and are therefore willing to accept and implement, it is arguably important to understand their current norms, values, and attitudes surrounding positive animal welfare [11,12]. However, a critical issue in this regard is the dearth of research directly examining farmers’ perspectives of positive animal welfare, rather than animal welfare more generally.

In response, this study uses qualitative interviews, informed by the free elicitation narrative interviewing approach [13], to explore farmers’ perspectives on positive animal welfare. This paper has three main aims. Firstly, it seeks to uncover whether farmers already engage in practices which (evidence suggests) may generate positive welfare, by exploring how indicators of positive welfare are constructed by farmers within their welfare-related discussions. Secondly, it aims to better understand what farmers consider is relevant to or indicative of positive welfare, by exploring the meaning farmers attach to positive welfare indicators. Thirdly, it seeks to understand whether positive welfare may arise from direct or indirect management practices, by exploring how farmers construct their role in the provision of positive welfare opportunities. Uncovering such insights contributes to the development of positive welfare indicators because it enables an understanding of what farmers think about in relation to positive welfare, and highlights what current practices, attitudes, and norms can be harnessed to effectively introduce positive welfare indicators at the farm level. As Hubbard and Scott [5] note, it is beneficial to involve farmers in the development of scientific techniques, as co-operation between scientists and farmers may support the promotion of higher welfare standards. As positive animal welfare is a newly emerging (and thus not necessarily well known) concept, there is a unique opportunity to explore farmers’ related perspectives and behaviours and co-construct positive welfare indicators, from the outset, in line with farmers’ language, constructs, values, and norms. 

## 2. Positive Welfare Indicators

Currently, there is no clear or agreed consensus within the literature on what positive welfare should entail or what makes a valid or reliable indicator of positive animal welfare. However, in line with the continued development and growing interest in this concept, several potential indicators for assessing positive welfare in farm animals have been proposed [2,3,14]. 

One of the most prominent indicators of positive welfare mentioned in the literature is positive affect (see [14,15,16]). Mellor [10] describes how providing animals with opportunities to engage in goal-directed behaviours which are rewarding can enhance positive welfare, and that such positive affective engagement can, in turn, be used as an indicator of an animal’s welfare state. Arguably, other positive welfare indicators suggested within the literature are often those which are believed to contribute to or promote the experience of positive affect, such as social interaction, play and environmental enrichment [14].

Play, for example, is presented in the literature as both an indicator of positive affect [7,14,16] and as a potential indicator of an overall good welfare state [17,18]. Similarly, the nature or quality of social interaction is presented as a potential indicator of positive welfare [3] and linked with positive affect [19]. Here, particular pro-social behaviours such as affiliation (e.g., synchronisation, group bonds) [20], care giving (e.g., maternal behaviour) [21] and social play [17] are thought to be relevant to generating positive animal welfare [19]. Environmental enrichment may also indicate positive welfare [2] as they can stimulate animals to engage in desired goal-directed behaviours [22]. Enrichments can include, for example, mechanical brushes which stimulate self-grooming in dairy cows [23] or the provision of straw which encourage pigs to play or engage in exploratory behaviours [24]. A positive human-animal relationship may also be relevant for positive welfare. For example, Edgar et al. [2] suggest that having positive interactions with stock keepers can support animal confidence. Enabling animals to exert some control over their environment (e.g., have autonomy or agency) has also been linked to positive affect [25] and proposed as a relevant aspect of positive welfare [26]. Closely related to agency, studies have proposed that further indicators of positive welfare could include providing animals with opportunities to exert individual preferences for thermal and physical comfort [2,27]. In addition, Edgar et al. [2] propose the importance of promoting health by “breeding for positive welfare”, as indicated by breeding decisions which support the long-term health and welfare of animals, along with a lack of mutilations and effective day-to-day management of health concerns. 

Notably, some of this research has involved farmers in the development of positive welfare indicators and frameworks. For example, Edgar et al. [2] first drafted a resource-tier framework of ‘good life’ opportunities for laying hens through consultation with experts (e.g., welfare scientists and welfare assurance schemes) before interviewing farmers to ascertain their opinions on the framework. More specific to positive welfare, Stokes et al. [28] described using focus groups and consultation interviews to collaborate with dairy and sheep farmers in the development of a framework for assessing positive animal welfare. This included several of the indicators mentioned above, such as positive social interactions and providing animals with some degree of choice within the system (e.g., autonomy). This research interest in working with and considering the farmers’ point of view exemplifies the increasing recognition amongst animal scientists that welfare improvement can often hinge on farmer acceptance or buy-in. Nevertheless, to continue the effective development of positive welfare indicators, further research on the perspectives of different farmers is needed, along with a clearer understanding of how they construct and give meaning to specific positive welfare indicators and the underlying factors of influence (e.g., farmers’ values, beliefs, attitudes, and social norms). 

## 3. Materials and Methods

This article is based on qualitative interview data collected as part of wider research exploring the perspective of livestock farmers and members of the public on positive animal welfare. This research explored several factors pertaining to societal understanding of positive welfare, including how citizens and farmers framed positive welfare, the aspects of positive welfare evident in their discussions, and the factors underlying and influencing farmers’ approach to welfare provision. This present article specifically focuses on the latter two aspects of this interview study.

### 3.1. Method

The free association narrative interviewing method, designed by Hollway and Jefferson [13], informed the design of the interview protocol. This interview method combines narrative interviewing techniques with the psychoanalytical principle of free association [13]. This means it encourages participants to tell stories and recount personal experiences, within which they have the opportunity to freely elicit factors which they attach meaning to. This ensures participants are unrestricted by any a priori research assumptions which can manifest in narrow or closed questions [13]. This allows the researcher to gain an insight of the participants’ perceptions, their representations of the self and the influence of wider social or cultural factors on their opinions [29].

The interview protocol of this study was centred around three focused narrative questions: (i) “Can you tell me about what, in your experience, is a good life for a farm animal?” (ii) “Positive animal welfare—can you tell me about what comes to mind when you hear that?” and; (iii) “Can you describe what motivates you to farm the way you do?” Although the purpose of this research was explained to farmers at the outset, no explanation of what is meant by positive welfare was provided here. The aim was to encourage participants to tell stories (in their words and from their point of view) related to their subjective perceptions of a ‘good life’ for farm animals and ‘positive animal welfare’ along with the motivations underlying or driving these perspectives. 

Although there is no singular way to conduct a narrative interview [30], the generally recommended approach is to first pose a singular narrative-inducing question (e.g., tell me your experiences of…) in order to encourage the participant to holistically recount their perspectives on a phenomenon. During this discussion, the researcher does not interrupt the participant or provide any additional prompts but passively takes notes on points of interest freely elicited within the participant’s narrative. Once it is clear this main narration has come to a natural end, the researcher can use these notes to ask more probing and detailed questions to gain a deeper understanding of the participant’s perspective and experiences [31]. 

Each of the three focused narrative questions were treated as a separate narrative opportunity and this approach was thus applied to each of the three main questions. Table A1, in the Appendix A, provides an overview of this structure along with some examples of follow-up probing questions, based on the topics mentioned by the participant in response to the initial narrative-inducing question. However, in most interviews the first question resulted in a lengthy response narrative by participants with numerous points raised which required further probing questions. The second question was similar in this regard, while the third question resulted in a shorter, more precise response from participants. This present article is primarily interested in responses to question one and three, as findings from question two have already been analysed in detail in a separate paper (see [32]). However, in instances where responses to question two are relevant to the aims of this paper, they are included in the findings. 

### 3.2. Sample

The population of interest was livestock farmers, from different sectors and with different farming systems (e.g., free-range poultry, zero-grazing dairy etc.). For this reason, the study used a purposive heterogeneous sampling approach to select a diverse range of participants [33,34]. In other words, farmers were not selected on the basis of being a representative sample but one that provided variety in type (e.g., different values, farming systems, etc.), so that as many different insights on positive welfare as possible could be gained. 

A variety of means were used to recruit farmers, including advertising on social media, referral from farm advisers and other industry groups, by directly contacting farmers via email or telephone, and attending farmer events (e.g., monitor farm meetings/discussion groups). This resulted in the recruitment of 28 farmers—13 dairy, 10 beef and sheep, two free range egg produces, two mixed farmers (i.e., had pig, poultry, beef and sheep) and one large pig farmer. 

The interviews were completed in-person, at the home or farm office of each individual between March and September 2018, in Scotland, UK. On average, interviews lasted 55 min. The majority of participants were male (86%), with a smaller number of female participants (11%) and one participant who preferred not to have their gender recorded. In line with the heterogeneous sampling approach, participants demonstrated a wide variety of different systems within each sector (e.g., organic or non-organic free-range poultry farmers, zero-grazed or pasture-based dairy systems, and indoor or outdoor-wintered beef and sheep). Further demographic information for each participant can be found in Table A2 in Appendix A. 

Before the commencement of the interviews, the research was approved by the Scotland’s Rural College (SRUC) social science research ethics committee. Participants were given a consent form, detailing the purposes of the research and how their inputs would be used, which they signed. Interviews were also audio-recorded, except for one participant who asked not to be, so detailed notes were taken instead. Interviews were continued until a saturation point, meaning no additional (i.e., ‘new’) data which had not already been encountered in prior interviews, emerged [35].

### 3.3. Data Analysis

All of the recorded interviews were transcribed in full (over half by the researcher who conducted the interviews and a smaller number by a professional transcribing service, which were then checked for accuracy by the interviewer) and entered into Max QDA; a software program for assisting qualitative data analysis. The constant comparison method, first described by Glaser and Strauss [36], was used to analyse the data. This involves reading each interview and categorising or ‘coding’ relevant sections according to the theme of the points raised. For example, codes which emerged in response to the question regarding a ‘good life’ for farm animals included physical comfort, health, social interaction, and positive affect (amongst several others). Once this initial coding was completed for each interview, they were compared between interviews and related codes and themes were grouped to form overarching superordinate themes. This ensured that the data generated was not influenced by any a priori definitions of positive welfare but were grounded in, or emergent from, the narratives of the participating farmers. These key, overarching themes provided insights into farmers’ perspectives on positive welfare and their current practices, as detailed in the subsequent section.

## 4. Findings

Although farmers were not familiar with the term positive animal welfare, it was evident that several aspects of their current management practices and attitudes towards animal welfare may provide positive welfare opportunities, and a base for their furtherance on-farm. In particular, the positive welfare opportunities of autonomy, play, positive affect, a positive human-animal relationship, social interaction, genetic selection and enrichment emerged as important and recurrent themes within farmers’ narratives. These concepts, and the meaning farmers attach to them, are presented in detail in the following sections. The final section describes the perceived role farmers’ consider they have in the provision of such welfare factors. 

### 4.1. Autonomy

Autonomy primarily arose within farmers’ narratives as providing animals with some degree of choice over being in (i.e., housed) or out (i.e., on pasture).

#### 4.1.1. Choice to Be in or Out

Pasture-based dairy farmers were the most common group to mention providing opportunities for their animals to choose to be in or out. However, the degree to which such autonomy can be provided is influenced by practical constraints, such as the design of the farm, the weather and the land type. The greatest level of free choice was noted by farmers operating a pasture-based robotic system (i.e., Dairy 6 and Dairy 12). Here, the milking herd have constant and complete choice over being inside or out:

*“Our cows have got free choice. If our cows want to go out, they’ll go out, if our cows want to stay in, they stay in”*.

(Dairy 12)

Such robotic systems also provide milking cows with free choice over when and how often to be milked. 

Those using a more traditional pasture-based system (i.e., grazed during summer, housed during winter, twice-a-day non-robotic milking) note they give their cows choice to be in or out some of the time, often due to the design of the farm:

*“The way we can graze one of our fields of paddocks, they can walk into the shed…Some, if it is a heavy night, they’ll go out, they’ll eat as much as they can, and then they’ll turn around and walk straight back in to the shed and lie down”*,

(Dairy 3)

or if the weather is bad:

*“Maybe it was raining one night, and the cows are not out but we didn’t chase them out. We just left them. We said we’ll tell you what, if the rain goes off and you want to go out, go out. If you don’t want to go out, don’t go out. And you see in the morning, all the cows were out. And that was no interference from humans, that was the cow’s decision”*.

(Dairy 5)

In addition, several pasture-based dairy farmers (and most beef and sheep farmers) noted that they looked to their animal’s behaviour to decide when to bring them in for the winter, allowing the animals to make that decision:

*“And quite often with the Autumn calvers, what we do is we let them out during the day, and once we’re finished milking, we’ll go and open the gates and they can come back in when they want to, and it is the day they are all standing at the gate waiting for you to open it, that is the day that you know they are not going to be coming out tomorrow. They kind of make that decision for themselves”*.

(Dairy 1)

Furthermore, most beef and sheep farmers argued that cattle and sheep are best raised outdoors in their ‘natural’ environment, with several of them arguing for the health and welfare benefits of out-wintering animals. To justify this, they drew heavily from stories of their animals preferring to be outdoors when given the choice, thus highlighting the value they place on animal choice and preference:

*“My experience is we have animals that have access to sheds but also have access to woods and even on the stormiest, horrible-ist night…. you’ll find the animals lying in the woods”*.

(Beef and Sheep 6)

The two interviewed egg producers both farmed free range, thus giving their animals full choice over being inside or out. However, both poultry farmers noted that many birds choose not to be outside:

*“Chicken by name, chicken by nature. They don’t go very far from their shed. They don’t, they cannot, they are too frightened to, even if you give them a lot of cover. Remember, all their feed, all their water, everything the hen needs is inside the shed”*.

(Poultry 2)

The two farmers who kept pigs (Pig 1 and Mixed 2), kept them fully indoors so there was no opportunity for animal autonomy in terms of choosing to be in or out.

#### 4.1.2. Interconnection Between Farmer Values, Farming System and Autonomy

A further interesting finding was an interconnectedness between farmers’ personal values, the way in which they farmed, and consequently, how they enabled autonomy. Farmers could be broadly grouped as strongly valuing either a highly structured or a loosely structured farming system. A highly structured system was evident in farmers who emphasised that better welfare was provided by closely controlling, managing and monitoring their animals:

“We’ve got a lot more control over their diet, over their health and welfare when they’re inside”

(Dairy 8)

Farmers with a loosely structured system emphasised that better welfare was provided by limited management intervention and essentially leaving the animal to ‘get on with its job’:

*“If that cow has 14, 15 calves on her own [over her lifetime]; that I never actually have to touch it, apart from tagging the calf, running the bulls and moving the calf from where she calves to the group…no intervention at all is ideal…she’s happy, I’m happy”*.

(Beef and Sheep 4)

Critically, differences in the way in which autonomy was perceived and enabled were evident between these different structures. Farmers who valued a loosely structured system often argued that limited interference on their behalf meant their animals had greater opportunities to exercise choice and autonomy:

*“They spend more time in the field. So they are 20 hours in the field out of 24. And then she is not interacting with anything but her herd mates, because there is no human interaction once you are in the field….they can eat when they want to eat. Walk about when they want to walk about. Drink when they want to drink. Sleep when they want to sleep”*.

(Dairy 10)

Conversely, animal autonomy in a highly structured system—where farmers valued the welfare benefits gained from having greater control of the animal’s environment—was more often discussed in the context of what could be provided within the boundaries of that system. For instance, providing plenty of space in housing so that animals could exert choice on where to lie or move around:

*“The bit they’re in are 14ft passages. So they’ve got enough space to run around and do whatever they want. They’re not having to barge by each other”*,

(Dairy 9)

or who to be near:

*“So the calves have got a nice big pen to themselves that only they can get in, so it gives them choice so there is not sort of any stress due to there being any forced interactions”*.

(Beef and Sheep 5)

Critically, issues may arise when the type of farming system does not correspond with the farmer’s personal values. This was notable in the narratives of the two poultry farmers. Both noted they farmed free range primarily because this is a publicly desired product. However, both appeared to value more structured and controlled farming systems. Consequently, they often highlighted the welfare issues that could arise within a free range system, due to the lack of close control and monitoring they would prefer:

*“There is a big misconception of what free range really is….in the middle of winter when it is terrible weather and they are plodding about in the muck and mud, there is a question of whether it is really higher welfare letting them outside or would it be better sitting them inside”*.

(Poultry 1)

Indeed, there were evident differences, across all farmers, in how loosely structured and highly structured farmers valued autonomy. The following narrative of a dairy farmer, with a loosely structured system, highlights how highly he values autonomy over other welfare considerations:

*“You get things like interaction between cows and dominance….there is all these questions around how that affects happiness and welfare, but I think…. having their own choice about what they do overcomes any concern about interaction”*.

(Dairy 10)

Conversely, the following dairy farmer who values a more structured system demonstrates a cautious approach to autonomy and gives greater importance to close management control:

*“I suppose you’ve got to watch with choice, because cows are like little children, if you give them access to sweeties they’ll get a very sore stomach, because they don’t stop, they keep going to get more. Aye, I suppose choice to an extent. Just let them go their own way but they still need management”*.

(Dairy 3)

Overall, autonomy and opportunities for choice was a frequent feature within farmers’ discussions of their animal husbandry practices. It was evident from the context of these discussions that farmers are cognisant of their animal’s preferences and will often look to ‘what would the animals choose to do themselves’ when making management decisions. However, there are differences in the extent to which farmers can provide opportunities for autonomy and indeed, individual differences between farmers in the value or relevance placed on providing such opportunities. 

### 4.2. Play

References to play were evident within farmers’ narratives. However, the majority of farmers construct play as a non-essential—a behaviour that did not require their direct input—as they perceived animals would engage in it anyway, regardless of what they do:

*“So, they [turkeys] like things going on….. just seeing things out and about. They don’t physically need to be playing with anything”*,

(Mixed 2)

and as something which is mostly reserved to young animals:

*“Well actually it wears off, you’ll see the hogs will skip and jump. The ewes will skip and jump, but you don’t see it very often….So it wears off with age. I mean there is no question it is much more obvious in young animals”*.

(Beef and Sheep 8)

The ‘non-essential’ status given to play leads to differences in terms of the role farmers perceive they have in promoting play and the relevance given to play as a welfare indicator. 

#### 4.2.1. Indirect Promotion of Play

When it comes to promoting play, the majority of farmers perceive their role is minimal as they construct play as an indirect outcome of other factors. Namely: (i) the innate natural behaviours of animals, particularly young animals, as presented above; (ii) good welfare provision (e.g., resource needs are met):

*“I would say that [play] is probably only necessary when all of the other needs are met….it is like us with leisure activities, if you have got everything else sorted and then you’ve got two hours where you have nothing else to do”*,

(Beef and Sheep 10)

and; (iii) social interaction:

*“And that goes again with social interaction, not being on their own. They should be allowed to play, to play with each other”*.

(Pig 1)

As such, most farmers do not perceive they have to actively promote or directly cause play as it is something which naturally arises from other management-based inputs.

#### 4.2.2. Direct Promotion of Play

However, it was evident that a small number of farmers provide inputs directed towards play:

*“We supply toys for the pigs to play with and the pigs will go over and they’ll root around the toys…so they’ll have a bit of plastic pipe or something they can chew. Then you’ll see them just chewing away with that. Just flicking it around as well”*.

(Pig 1)

*“We sometimes just leave things for them [calves] in the pen to look at, like a tyre….and they are all over looking at this tyre thinking what the bloody hell is that. And you take the tyre away and then you maybe leave a bucket….and they are playing with this bucket and they think ‘oh this is great’. We do that with horses. We buy your dog a toy. Why are calves any different”*.

(Dairy 5)

Importantly, as noted above, the provision of play objects was almost exclusive to some dairy farmers and the pig farmer. Beef and sheep farmers did not generally provide things for their animals to play with as animals were often noted to naturally make use of objects in their outdoor environment (e.g., branches of trees) or play with one another in their social groups. Interestingly, one farmer noted differences in the play behaviour of lambs when brought indoors to a less ‘enriched’ environment:

*“So when we’ve ended up with lambs in the shed, finishing lambs in the shed around Christmas time, so they’ll probably be seven months old… and you’d see them playing out in the field but when you’ve brought them in to the shed, you’ve got less. They want to play with stuff, they do, and we don’t [provide anything]. There is not a lot of interest for them”*.

(Beef and Sheep 9)

Thus, the direct promotion of play is a management input only a small number of farmers perceive is necessary or worthwhile to engage in.

#### 4.2.3. Play as a Welfare Indicator

Regardless of whether farmers directly promote play or whether it is an indirect outcome of other welfare provisions, it was notable that many farmers use play as a welfare indicator. Specifically, play is seen to indicate that welfare provisions such as social interaction are adequate:

*“If social interaction is right, physical comfort is right, you will get play”*,

(Beef and Sheep 1)

and that overall animal well-being and welfare provision is good:

*“I would say that play is the outcome of welfare. I mean can I say, I think it is a correlate, at least, I think it is a strong correlate but I can’t say it is the most important thing… the best I think you could say of play is that it is a correlate of well-being, because I think it is an expression”*.

(Beef and Sheep 8)

Overall, actively promoting or causing play is not something farmers perceive they are required to do (as animals do it anyway). Rather, the majority of farmers construct play as something which will occur once other welfare needs (e.g., resource provision, social interaction) are met. However, the value farmers give play is more complex than simply saying they do not value it because they do not directly seek to cause it. Instead, they value it as something which is ‘nice-to-see’ and as a welfare indicator to signal that other welfare requirements of the animal have been met. 

### 4.3. Positive Affect

Positive affect emerged as a strong theme within the findings, with farmers freely eliciting numerous stories of how, when and why their animals experience positive affect. However, farmers’ construction of positive affect is complex with several different types being discussed and often used interchangeably.

#### 4.3.1. Contentment

Contentment is, by far, the most frequently mentioned affective state and the one farmers gave the most importance to. To participating farmers, contentment means that an animal is at ease with their environment and that their resource needs are appropriately met. Findings reveal that farmers assigned ‘contentment’ the role of something they sought to induce or achieve through their management-based inputs and the role of an animal-based output they looked to, to gauge the welfare state of their animals and thus, assess the efficacy of their own management practices:

*“If I feed my cows before I go for my tea at night, come back out from my tea and they have eaten as much as they possibly can, they are all lying down. So basically if I go back out from my tea and I push the silage in and very few of them would get out of their cubicle and come and eat silage and they are all quite happy and lying down. That is what I am aiming for”*.

(Dairy 3)

‘*Chewing their cud*’ was, far and wide, the most frequently used phrase amongst farmers of ruminant animals in this context. To say their animals were ‘chewing their cud’, was to holistically capture and convey all the elements of what contentment meant to them and its role as a welfare indicator. Namely, that their animals ‘want for nothing’ and are at ease as a result, thus indicating to the farmer they have effectively fulfilled their duty of care to their animals. 

However, farmers often used contentment interchangeably with happiness. On numerous occasions, even when directly asked to clarify their meaning, farmers presented ‘happy’ to mean the same as ‘content’ and vice versa:

*“You know cattle and sheep are happy [when] you go into a shed and they are all quiet and they are lying down and sitting chewing their cud, and content. Content is what I would say rather than happy”*.

(Beef and Sheep 10)

Overall, farmers highly value ‘contentment’ as the primary and most important affective state for animals to experience and the affective state they most desire to induce. They rarely distinguish between happiness and contentment. Instead, they appear to assign the same characteristics of animals being at ease, animals’ resource requirements being met, and farmer satisfaction (on seeing such contentment) to the context of contentment and happiness. Consequently, it is perhaps more relevant to refer to this desired affective state as happy-content, where the primary indicator of such a positive state to farmers was animals eating, lying down or demonstrating a general state of ease or relaxation:

*“If it is comfortable and happy and chewing it’s cud. Eating plenty and lying down plenty. If it is chilled out and relaxed and healthy, then I’d say it is a happy cow”*.

(Dairy 9)

#### 4.3.2. Happy-Energised

Nevertheless, there were some exceptions to farmers construct of ‘happy-content’ in the presentation of a further positive affective state of ‘happy-energised’. Rather than the calm, at ease or passive behaviours of happy-content animals, farmers characterised happy-energised as animals demonstrating much more expressive behaviours such as play, skipping or high-kicking:

*“What is a happy cow, well one that does that, runs off down the field with its tail in the air and jumps about. Like it is quite, there are physical behaviours”*.

(Beef and Sheep 6)

As the above narrative alludes to, discussion of happy-energised indicators were primarily within the context of animals being turned out to pasture (either a new field or being released from winter-housing), interacting with one another or engaging in play behaviours (particularly in young animals). However, unlike happy-content, which farmers actively sought to create, happy-energised was a behaviour which farmers did not perceive required an active role on their part. Rather, they enjoyed seeing it and evidently recognised the enjoyment their animals derived from such experiences but it was not something necessitating managerial input; animals would freely elicit such behaviours themselves.

#### 4.3.3. Pleasure

More broadly, there were numerous instances within farmers’ narratives where they described experiences which they considered were pleasurable for their animals. For the most part, this included the apparent pleasure gained from their environment and food and, to a lesser extent, play and autonomy. Pleasure from environmental factors included engaging with environmental enrichments, such as automatic brushes in dairy cows or litter for dust-bathing in poultry:

*“That then is them just having a bath and enjoying themselves…it is good when you do see the birds enjoying the sun and scratching about”*,

(Poultry 1)

enjoying natural features of their environment, such as good weather and dry pasture:

*“So a lot of the time it is just them lying down and enjoying being outside in the sunshine”*,

(Dairy 6)

or exploring novel environments:

*“And we have to laugh at times…we’ve got double gates going into an area, and it’s only about the size of this kitchen and we’ve got two pallets of sawdust…and honest to Christ, see if you leave that gate open for two seconds, the cows are straight in there…they are really clever”*.

(Dairy 5)

Farmers also noted the apparent enjoyment their animals experienced from eating particular foods, with some even noting the potential pleasure-related benefits of offering food choices and variety:

*“The fact that they clearly do like to eat a lot of things apart from rye grass and clover suggests that they see something in it and I don’t know whether it is just simply variety or looking for some mineral nutrient, but it might be nice for them”*.

(Beef and Sheep 8)

Closely connected to the previous two sections on autonomy and play, it was evident within several farmers’ narratives that they perceive their animals gain pleasure from such experiences. Play, from a farmers’ point of view, is an evidently pleasurable experience because of the energised and expressive behaviours animals display. Similarly, autonomy is constructed by some farmers to enable positive affect because it allows animals to engage in behaviours they value, such as exploration and social interaction:

*“Well happy is just having the freedom to know you’ve got everything that you need and you know you can go and have a wander around and see your surroundings; there’s other animals and other fields to see. We turned out 85 calves yesterday and you’d see them skipping and jumping and they stood and grazed for half an hour and then they were skipping and jumping again”*.

(Beef and Sheep 3)

Thus, overall, a multifaceted range of affective states are evident within farmers narratives with ‘happy-content’ emerging as the most highly valued and actively promoted form of positive affect. However, a discussion of affect in this context would not be complete without detailing its strong connection with productivity and animal performance.

#### 4.3.4. Positive Affect and Productivity

From a farmers’ point of view, an animal’s productiveness is a reflection of its happiness:

*“I suppose a happy cow has got to be producing milk. If there’s something wrong with it, if it’s off its milk [yield has reduced], from our dairy point of view we have that as an indicator. If it’s lying down, chewing its cud, happy with life, walking evenly on four feet [she is well]”*.

(Dairy 9)

As such, for farmers, the productivity output of their animals is objective evidence that their animals are experiencing positive affect. Indeed, the relative value farmers place on ‘happy-content’ as a welfare indicator appears to arise from its interconnection with animals being stress-free and healthy, both of which are constructed by farmers as primary drivers of productivity. In other words, the promotion of positive ‘happy-content’ states is partly motivated by a desire to negate negative stresses or health-issues:

*“For the cow to be contented, it’s much better because it’s less stressful, therefore she’s more content, therefore it’s not going to cause any issues with the calf that’s inside her, etc.”*.

(Beef and Sheep 7)

Nevertheless, regardless of the direct or indirect role of farmers in promoting positive affect, it is evident within farmers’ narratives that they are cognisant of animal emotion and that they look to their animal’s behavioural and affective expressions as an indicator of how it is experiencing its environment and their management inputs. 

### 4.4. Human-Animal Relationship

The nature and quality of the human-animal relationship—how the farmer interacts with their animals and how animals interact with the farmer—appears to be intrinsically personal to each farmer, with their individual values and life experiences influencing how they navigate this. Nevertheless, the findings reveal some key themes in this regard, consistent across individual farmers and farming sectors.

#### 4.4.1. Calm, Respectful Handling

First and foremost, farmers emphasise the importance of calm, respectful handling and developing familiarity with handlers as a critical husbandry skill; one which positively affects the quality of the human-animal relationship:

*“Quietly, in a friendly sort of way and for almost all of the year I am able to be respectful and appreciative of them; your patience wears a bit thin over lambing”*.

(Beef and Sheep 8)

*“Our cows are very quiet, and I would base that on the fact they they’ve been brought up quietly…we just work quietly with them and I think we feel that that is just the best way”*.

(Dairy 11)

This construct was often illustrated further in farmers’ discussions of their handling preferences. For instances, some sheep farmers felt limiting or not using dogs to herd sheep enabled more positive human-animal interactions:

*“And I think that if you round up sheep with dogs and bring them into the yards and have dogs in the yards keeping them going…all you’re doing is winding them up”*.

(Beef and Sheep 9)

Interestingly, several beef farmers detailed the use of squeeze crates and curved races, based on the work of Temple Grandin, as a means to reduce stress during handling:

*“It’s a pneumatic crush so it puts pressure on them. I suppose it’s Temple Grandin’s theory that if we put a little bit of pressure on them they feel contained…so they seem to be more comfortable”*.

(Beef and sheep 3)

*“I have the curved race… stress levels reduce on human and animal hugely”*.

(Beef and sheep 7)

In addition, dairy farmers who have transitioned to robotic milking detail their experiences of improvements in the quality of the human-animal relationship as a result. Mainly, because they are no longer having to herd or actively move cows (e.g., to collect and bring in for milking), the cows appear more relaxed during human interactions:

*“You realise when you move to a robotic system…how much time you spend chasing cows to the milking parlour…they get moved a lot. Whereas these ones, the idea is to move them as little as you can, and there is definitely some individuals who have settled on to the robot system…we had one with a sore foot about a month ago, and you were able to lift it like a horse in the shed, walk up to her, run your hand down the side of her leg and she lifted her foot for you. They are just not expecting you to try and move them somewhere”*.

(Dairy 6)

Overall, farmers highly value the importance of minimising stress on their animals and this, in part, underlies their motivation to handle their animals in a calm and respectful way.

#### 4.4.2. Knowledge of Animal Characteristics

Knowing the characteristics of individual (if not all) animals within the herd, is emphasised by several farmers as important for the quality of the human-animal relationship, particularly amongst dairy farmers:

*“I know every cow out there. If something walks in… you know straight away if there’s something not [right], it sounds silly; there’s 300 cows and you can’t possibly know them but you do, you spend every day with them”*.

(Dairy 9)

Indeed, many farmers demonstrate a strong sense of pride in being able to distinguish individual animals within their herd and their unique characteristics. They often see this ability as a critical hallmark of a good stock-person and one which can determine how they specifically interact with individual animals (e.g., more quietly). Thus, possessing knowledge of the habits and personalities of individual animals, where possible, is something several farmers emphasise and give weight to. However, others do note that this becomes more challenging with larger stock numbers and highlight the benefits of technology (e.g., mobility trackers) in this regard. 

#### 4.4.3. The Animal-Human Relationship

A further noteworthy facet of the human-animal relationship is the effect of animals on their human carers. There are numerous instances within the narratives of participating farmers detailing how their animals have influenced their human experience on the farm. This was particularly evident in the context of farmers discussing their animals’ affective experiences. Namely, when farmers recounted stories about their animals engaging in apparently pleasurable activities and displaying their enjoyment, they nearly always accompanied it with a discussion of the personal enjoyment they experienced from seeing this. In other words, positive affect in animals appears to engender positive affect in farmers, as illustrated in the following narratives:

*“I like the summer because I move my cattle everyday….I like the physical interaction of the cattle…where you go to a group of 120 cattle and those cattle see you arriving and they all get up and wander over to the corner and wait for you to arrive to roll back that reel, and them to walk through….all you are listening to is the rip of that grass…and those cattle just as content as they could possibly be….it does make me feel good”*.

(Beef and Sheep 6)

*“There is nothing better than seeing the calves interacting and playing outside. And lambs, you know, when they are a week or two, maybe at the two week old stage, you can’t beat watching lambs racing about the field and just being in their natural environment”*.

(Dairy 11)

Thus, overall, it is evident that farmers and their animals have an influence on one another, with farmers recognising the impact they have on their animals’ daily lives through their handling and management practices, whilst also, often unconsciously, highlighting the impact the behaviour and affective states of their animals has on them.

### 4.5. Social Interaction

Social interaction is important to farmers. All ubiquitously stressed that farm animals are gregarious and therefore should not be kept alone or prevented from interacting socially. For many farmers, preventing animals from socialising or keeping them alone was a welfare issue:

*“They get most stressed when they are on their own. That is the thing they dislike more than anything in my view”*.

(Beef and sheep 10)

*“Isolating an animal, …if you isolate anybody, human or animal, …it’s not the best environment for their mental well-being. And pigs are very sociable. I think if you put a pig in a pen on its own it would just, it wouldn’t be happy. We would only put them on their own if they are sick”*.

(Pig 1)

As such, keeping animals in social groups is considered a basic welfare requirement by all farmers.

#### 4.5.1. Minimising Negative Social Interaction

Farmers primarily consider social interaction as an animal-based output which, similar to play, animals will naturally engage in. Nevertheless, farmers present themselves as having some influence on social interaction, where their management-based inputs can impact the nature of interaction. Here, the majority of farmers construct and confine their management role to the reduction and prevention of negative interactions such as dominance or bullying, usually by separating animals into different groups:

*“You would probably separate the older more dominant animals and put them together and let them fight it out and take away the smaller, younger ones, and put them together. But they will still come up with a hierarchy but it might not be quite as dominant and subordinate as it would be if they were all mixed in together”*,

(Beef and Sheep 10)

designing living spaces so subordinate animals can get away from dominant animals:

*“This is one of the benefits of these multi-tier type sheds [as opposed to flat-deck poultry housing]…the dominant birds tend to take up residence in the top, they will have, you know, their pecking order”*,

(Poultry 2)

or not unnecessarily mixing animals between groups or herds:

*“We have multiple herds, so it is possible for us to change the herds, to mix up the cows in the herd. We try as far as possible not to do that, as you definitely get dominance”*.

(Dairy 10)

*“You don’t want to potentially mix pigs, which will create stress”*.

(Pig 1)

One dairy farmer even noted measuring the impact of such negative social interactions on their cows:

*“Some weeks when you put the six or seven new cows in….the cell count you’ll see will have a little spike and it takes two or three days to come back down again, and that is them just sorting themselves out in the dominance line, or those cows have just come in and are maybe just worked up a little bit”*.

(Dairy 6)

Thus, it is clearly evident that farmers are cognisant of negative social interactions between animals, are aware that their actions can result in negative social interactions and thus seek to minimise both.

#### 4.5.2. Supporting Social and Maternal Bonds

Beyond the focus on negative social interactions and their preventions, there was some evidence of enabling positive social interactions. Mainly, this centred on the support and maintenance of social bonds. Several dairy farmers, for example, aimed to raise their replacement young-stock (e.g., calves and heifers) in small-groups, enabling them to transition these animals together into the main herd so they could maintain their existing social groups:

*“Some of the cows now will stay in batches and you’ll see that all the 100s kind of hang about together and the 200s, and they were heifers that were all reared together and they know each other”*,

(Dairy 12)

or by splitting larger herds into smaller herds:

*“I think we will end up with a young cows group…we will have this as a heifers group and the next shed as our high performing group”*.

(Dairy 6)

Supporting positive maternal bonds was also particularly important to beef and sheep farmers:

*“The parent-offspring bond in animals is pretty strong…when we have sucklers…we don’t tend to wean until the new calf comes along, so it is sort of natural weaning”*.

(Mixed 1)

*“What sheep want is food and they want their lambs and the lambs want their mothers…I’ve observed that they want to hang around the animals that they are familiar with and grew up with…they stick together in the groups that they’ve known from when they were born or kept together”*.

(Beef and Sheep 8)

In particular, they often told stories of specific ewes or specific cows whose high performance they attested to their excellent mothering abilities, such as in the following narrative:

*“So the ewe that started taking the flock higher [i.e., improved performance] because we used a lot of sons of hers….when we shed her lambs to drench them…..she is waiting at the end of the race. She’s not gone back to the field. She is waiting at the end of the race and she gets one and she waits and gets the other one….and we’re claiming that she’s milkier or has better growth potential….but it’s her behaviour that has driven that I think. So, I think there is a lot more in the ewes’ behaviour than what we’re giving credit to”*.

(Beef and Sheep 9)

Overall, farmers highly value social interaction and see it as a basic requirement and need of their animals. As such, farmers predominantly view social interaction as a natural behaviour which does not require much management or input from them, beyond intervening to minimise negative social interactions. Nevertheless, they are cognisant of the benefits of positive social interactions to their animals and some seek to support this through the maintenance of social bonds and limited mixing of animals between groups. 

### 4.6. Genetics

The farmers in this study presented genetic selection as a critically important aspect of their management decisions, one which they felt could impact the longer-term welfare and health of their animals. Although the importance placed on genetics was shared across all farmers, it was evident that different farming sectors were motivated by different genetics-related goals. 

#### 4.6.1. Welfare Aims Amongst Beef and Sheep Farmers

Beef and sheep farmers, in particular, saw thinking long-term about the type of animals they wanted to breed as a central part of welfare. Three main genetics-related goals emerged within this farming group. First, they emphasised the importance of: (i) selecting for calmness:

*“So the long term goal of that is to have a flock of ewes here that are happy in their surroundings, in their environment, they’re not stressed….and an important aspect of that I think is that the ewe’s calm enough…that is a state of mind thing I think for the sheep….there is a breeding in the sheep”*.

(Beef and Sheep 9)

This genetics-related goal was motivated by wanting to reduce the overall stress their animals experienced while being handled or interacted with. This often led to very specific preventative breeding decisions, such as using polled genetics to avoid the stress caused by de-horning calves:

*“We’ve not been a fan of dehorning calves, purely because it’s an extra stress on the cow…so we’ve been Angus breeders for 20 years, 25 years, so now they’re polled, even the Limousine genetics are all polled. So, just one less stress for the cows and the staff as well”*,

(Beef and Sheep 3)

and removing ‘difficult’ animals from the herd to improve its overall ‘calmness’:

*“I’ve got a very active policy here that anything that annoys me at all for any reason is just gone….anything that is very flighty, anything that causes me hassle…which means, and I can see it, the herd is getting easier and easier to manage, they’re getting quieter…everything is calming on its own….I’m not having to go and pull out big calves….so surely, that’s what welfare is”*.

(Beef and sheep 4)

Closely related to this, was beef and sheep farmers’ second genetics-related goal of (ii) selecting for ease of management and health. Here, the aim was to produce animals which needed limited intervention (e.g., could calve unassisted):

*“We try and use easy-calving strains”*,

(Beef and Sheep 3)

and had the behavioural and health traits required for the farm’s longer-term performance and sustainability.

*“Welfare is also an issue if you find that you’ve got to be intervening all the time using antibiotics, fixing things, bad feet. Surely you should be able to breed all that out so the cow looks after itself, which means that’s the best welfare”*.

(Beef and Sheep 4)

An additional goal of beef and sheep farmers was to (iii) breed to suit the farm system and environment, as opposed to performance-based factors alone. Here, farmers felt that animals would perform better if their breeding suitably equipped them to thrive within the specific topography of the farm or within the farm’s management system:

*“So the reason we’ve done well I think is because our Luing cows spend their life here thinking, ‘Life’s a breeze! We go out in the summer and there is abundant grass and we get pregnant straight away and job’s a good’un’. Whereas our Simmental cows, you put them out and they sort of stand at the gate wishing they lived on a better farm, so that is the difference…at the moment we’ve got a drive on to try and change the type of Simmental we have. I think the Simmental has a good role to play [so] we are trying to head for and get a low input Simmental”*.

(Beef and Sheep 9)

In sum, beef and sheep farmers thought long-term about the type of animal they wanted to breed. They emphasised the importance of selecting for calmness to produce animals that experienced less stress, needed less management intervention and were genetically equipped with the mental or behavioural traits needed to perform well within the specific conditions of their farm.

#### 4.6.2. Welfare Aims Amongst Dairy Farmers

Amongst participating dairy farmers, using genetics to overcome welfare or health issues experienced in the past or to prevent potential future health issues, appeared to be the main goal of their genetics-related decisions. For instance, they used genetics to address lameness and mobility issues:

*“We have dealt with lameness by cross-breeding and making sure cows have got black feet”*,

(Dairy 10)

to support health:

*“Even before they are born…you are selecting bulls for management traits which are going to lead to cows which are going to last longer, which are going to have fewer problems,…keeping that focus on health and welfare right throughout their lives”*,

(Dairy 1)

and to overcome the ‘bull-calf issue’ (i.e., lack of a market for bull calves and negative public perception of their culling) by using sexed semen:

*“We’ve gone down the route in the last, just kind of nine months now, of just using sexed semen…it is nice knowing that in nine months’ time, I’m going to get a heifer calf rather than at the end of it you get a bull calf that is, to us, no use really”*.

(Dairy 9)

Similar to beef and sheep farmers, breeding animals which suit the system of the farm is also an important genetic consideration for dairy farmers:

*“The ultimate, this is a question I guess, could the ultimate driver of positive welfare be having the right genetics for your phenotype? And in actual fact I would suspect that is the case, because I need cows that can handle all the rubbish and poor management that I throw at them”*.

(Dairy 10)

The longevity of their cows also emerged as important. Many farmers noted concerns that productivity-based selection of the past (e.g., higher milk yield) had produced cows which could only do a small number of lactation cycles before health problems occurred (e.g., lameness or mastitis):

*“A long life. There are too many animals that go away too early now. That is probably the way we have bred animals though isn’t it”*.

(Dairy 4)

Consequently, several farmers emphasised the importance of milk yield over the animal’s life-time (rather than per lactation) and thus selected on factors needed for cows to have a long life (e.g., mobility and health).

*“We’d rather reduce the litres and have a cow that lasts longer and gets in calf, rather than have a big showy cow that will only last two or three lactations… [and that comes] partly from breeding, certainly for more ligament strength…..a lot of cows coming on that don’t make it that long because udders are hanging off them, dragging on the ground is no use, so there is definitely a bit of breeding in it”*.

(Dairy 3)

Interestingly, dairy farmers stressed genetics as being a key driver of improved health and welfare in farming in the future, with several commenting on the potential of gene editing in this regard and two farmers noting they genetically test some of their young-stock to inform their breeding decisions. The ubiquitous use of artificial insemination amongst participating dairy farmers may play a role here; having such detail on the genetic characteristics of particular bulls appears to make them more cognisant of the health and welfare implications of genetic selection, when often, trade-offs have to be made and particular genetic traits have to be prioritised:

*“We quite often find that we don’t end up using the bull with the highest [scores] for milk yield or the highest fat and protein yield because there is one that has got very good feet and legs or one that has got very good udder confirmation or something like that”*.

(Dairy 1)

Overall, dairy farmers emphasised the importance of using breeding decisions to improve health, minimise previously experienced welfare issues and ensure animals suit the farm environment and can lead a long life. Such detailed discussion demonstrates the value farmers place on genetics, primarily as a means to overcome negative health issues, and in doing so, improve welfare.

#### 4.6.3. Welfare Aims Amongst Pig and Poultry Farmers

The discussion of genetics in terms of health and welfare factors was less notable amongst pig and poultry farmers, potentially as breeding in this sector is generally carried out by specialist breeding companies. However, one poultry farmer, similar to dairy farmers, highlighted the potential for genetics to overcome welfare issues, in this case feather-pecking, but rooted the challenges of this in terms of its effect on productivity:

*“We would like to see a genetic change to the hens so eventually they would, we would like to breed this beak [tip] off…the problem is, you could breed that beak off and lose 50 eggs, no use to me. We’re wanting the unachievable perhaps…unless, you as a consumer are willing to give me £3 a dozen for the eggs. But you’re not”*.

(Poultry 2)

Overall, genetics-related decisions are a central aspect of farmers’ management practices and one which they place considerable importance on. Indeed, as presented here, genetics are often used to reduce welfare or health concerns, where the overarching end-goal is to negate issues and improve performance. However, it is notable that many farmers recognise that selecting on productivity-based factors only (e.g., growth or yield) can have adverse health and welfare consequences. Thus, they engage in genetics practices which can indirectly benefit performance and productivity by producing animals which are calmer, are tailored to the specifics of the farm environment, require less intervention, have a longer life, and are, above all, healthy.

### 4.7. Environmental Enrichment

Overall, farmers place little emphasis or importance on environmental enrichment. This is because, for the most part, farmers perceive that their animals are adequately enriched by the typical environments on a farm. For instance, beef and sheep farmers believe that access to the outdoors provides them with appropriate enrichment, mainly as this enables their animals to interact socially, engage in play behaviours, graze on various plants in hedgerows or in fields, scratch or rub themselves on fence posts or tree branches and explore their fields and surroundings. Consequently, beef and sheep farmers see little requirement for or additional benefits from providing enrichment objects to their animals:

*“The animal is much happier outside and we manage our animals in such a way, they get moved every single day 365 days of the year, my cattle are behind an electric fence and that electric fence gets moved every single day…it is moved every single day, so every single day they are on to a fresh piece of pasture”*.

(Beef and Sheep 6)

*“Sometimes bulls and things will have a bit of a rub on and things like that, so I think it is important that they do have stimuli of some description, even if it is just a branch of a tree….I wouldn’t say it is cruel to not give them rubs, I would say that is an extra add on rather than an absolute necessity”*.

(Beef and Sheep 10)

*“I think that certainly cattle, as long as they’ve got interaction with their own, on a day to day basis, they’ll not be bored as such, or well ours have got plenty of chance to go and rub and have a scratch and stuff like that. They’re quite, my take on cattle is that they are quite routined”*.

(Beef and Sheep 5)

Nevertheless, dairy farmers, particularly those who have a zero-grazing system, discuss the use of automatic brushes as an enrichment tool for their animals. In most cases, they value the importance of such enrichment objects because they observe and recognise the pleasure and enjoyment their animals get from using them:

*“We’ve got brushes as well, the automatic brushes…and they absolutely love them. And you can tell if they’ve been dry [i.e., not milking] and they come in to the parlour again that is probably one of the first things they go and do…so it must be quite enjoyable for them”*.

(Dairy 11)

One farmer even noted how observation of cows’ use of automatic brushes could highlight other issues:

*“The other thing though with brushes, I have a friend who sorted the ventilation in the shed, and after that he said the cows weren’t bothering to use the brush and he reckons that because the air is cleaner the cows are staying cleaner, and they are not needing to go and brush themselves as much”*.

(Dairy 3)

Enrichment, as a management-based input which farmers provide, is most evident in the narratives of pig and poultry farmers. Free range egg producers emphasised the importance of providing litter to hens as an enrichment tool to enable them to dust-bathe:

*“The ultimate enrichment for hens is litter…. my hens display all the traits of being perfect, with their feather cover, and they have tremendous litter on the floor. And litter is what it is all about, for a hen, litter is what it is all about, it is like giving that dog a bone. It is just what they want and they display it immediately…there will not be an inch left where there is not a hen in it. So they must like it”*.

(Poultry 2)

In addition, enrichment objects were often seen as a way to prevent negative interactions, such as feather-pecking:

*“I have big square bales…..and they’ll jump up and down on that and run around that….if they do get bits of feather pecking, I’ll go in with maybe cabbages or something. Throw some cabbages down and they can peck away at those so I do have to make sure their environments are rich. I think a bored turkey can be quite a dangerous thing. To each other”*.

(Mixed 2)

The large commercial pig farmer also discussed the provision of toys to pigs in slatted housing and noted the benefits of such enrichment objects for the pigs:

*“I think when toys were first brought in, everyone thought that they were stupid, because they thought what the hell are we doing this for. But you do see them playing with them. And I think that is maybe not a bad thing”*.

(Pig 1)

Overall, enrichment objects primarily appear within farmers’ narratives as something which only animals kept indoors require, those which live most of their life outdoors are perceived as being adequately enriched by being in their ‘natural’ environment. Consequently, although farmers often discuss and tell stories of the enjoyment and pleasure their animals get when interacting with environmental enrichments, they do not generally place as much importance on this as other factors such as social interaction or autonomy (which are often presented as means of enrichment in and of themselves).

### 4.8. Positive Animal Welfare from Direct or Indirect Management Practices

A noteworthy finding is how positive welfare opportunities arose from both the direct and indirect management practices of farmers. The context of farmers’ animal welfare discussions reveal that there were some welfare indicators they actively seek to provide and there are others which they consider are ‘nice-to-see’ (i.e., are not essential or high priority). Resource needs, health-related factors and stress reduction often fall into the category of intentional management provision, but animal-based inputs and outputs often fall into a category of ‘nice to see’. This is potentially due to the importance farmers place on reducing stress and fulfilling their duty of care by providing an environment which appropriately meets the resource need requirements of their animals (e.g., nutrition, physical comfort, space) and supports their health. Nevertheless, it was evident in farmers’ narratives that positive welfare indicators arose from both direct and indirect management practices. 

For example, autonomy was directly enabled by farmers who provided their animals with the choice to be in or out, whilst also being indirectly supported by the provision of space and housing design and indirectly affected by the farming system the farmer developed based on their personal values (e.g., loosely structured or highly structured). Similarly, positive social interactions were directly supported by farmers’ actions to minimise negative social interactions (e.g., dominance) and support social bonds (e.g., not mixing groups), whilst also indirectly supported by space allocation and housing design. Furthermore, there can be differences in farmers’ direct or indirect management practices within a specific welfare indicator, with positive affect being the primary example. Here, all farmers directly sought to induce contentment in their animals, through the provision of desired resource needs. However, more high-arousal emotions, such as pleasure and happy-energised, were indirectly enabled by other management practices such as the provision of enrichment objects or following a change in environment (e.g., turned out to pasture in spring time). 

Such insight and knowledge is important because factors which arise from direct inputs are arguably those which farmers consider the most relevant and critical for their animal’s welfare. This is pertinent for the development of positive welfare indicators as they reveal what farmers are currently doing, the welfare indicators they are interested in measuring, and what welfare outcomes they particularly value. Farmers may be more likely to accept positive welfare indicators if they are in line with direct practices they already focus on or can arise indirectly from the other management inputs they value. 

However, these findings also reveal that farmers do not look at these factors and welfare indicators as singular unrelated entities but see them as an interconnected whole which support and influence one another to impact their animals’ well-being. For instance, farmers wrap social interactions up with other factors such as autonomy and play, perceiving that if animals have the freedom to move around and choose who they want to be with, then they can experience positive social interaction, and if they experience social interactions then they are more likely to play. Similarly, if animals are free to socially interact and have all their resource needs appropriately met then they are more likely to experience positive affect. As the below narrative illustrates, farmers’ construction of welfare indicators is highly interconnected and are seen as mutually inclusive:

*“I think social interaction is very important. I think play comes as part of social interaction, so I don’t think play is critical, so you will see that as play being; if social interaction is right, physical comfort is right, you will get play. Which is a good indicator that they are good”*.

(Beef and Sheep 1)

Thus, the findings of this study reveal that farmers construct such positive welfare indicators to be indirectly supported and enhanced by each other. 

A final, noteworthy finding in this regard, is the context-dependency of welfare indicators to farmers. When discussing different aspects and indicators of welfare, many farmers commented on how their specific importance would change depending on the context of the situation. For example, as the farmer below describes, social interaction as a welfare indicator would increase in importance if an animal was kept alone:

*“If you are looking at it in the fact that there’s an animal on its own, then that becomes number one above anything else; it needs to be with someone else. But if it’s looking between a group of ten or a group of 30, it probably doesn’t mean a lot. You should never leave an animal on its own”*.

(Beef and Sheep 9)

Furthermore, as the farmer below explains, the context of perspective, whether it is the animals’ or the farmers’ perspective, also influences the value placed on particular welfare indicators:

*“Well if you give them choice, they wouldn’t exercise [but]….I think that’s crucial for their fitness. But that’s me. If the context is what the cow thinks, the cow couldn’t give a toss about their fitness”*.

(Dairy 10)

Such findings reveal the temporality of welfare indicators to farmers. In other words, farmers will often gauge the importance of one welfare indicator against another or against the specifics of the context in which they are in; often, trade-offs have to be made. In addition, farmers continuously stressed the importance of adapting and tailoring their welfare approach and husbandry practices to the specifics of their farm, the type of animals they have (e.g., personality characteristics and breed) and their own personal vision and ethos. Considering all of these factors, it is arguably best to construct farmers’ focus on particular welfare indicators as fluid; altering and responding to the needs of different seasons, the specifics of different management systems, adapting to external forces (e.g., market changes) and tailored to the typography and characteristics of their particular farm. In sum, farmers engage in both direct and indirect management practices which may support positive welfare but the nature of that engagement is influenced by the unique characteristics of their personal situation, values and farm. 

## 5. Discussion

To support the development of positive welfare indicators, this paper set out to explore whether and how farmers describe positive welfare indicators within their current practices, the meaning they attach to them and how they construct their role in the provision of positive welfare opportunities. Notably, findings indicate overlaps between the positive welfare literature and farmers’ current practices. For example, farmers appear to: value and, to some extent, support positive social relationships [3]; enable a positive human-animal relationship through respectful handling [37]; look to play to assess an animal’s welfare state [7]; provide animals with some choice within their environment [25,26,38] and link high-arousal positive affective states (e.g., high-kicking) with situations of contrast (e.g., moving to a new field) [39,40]. However, when looked at as a whole, it is critical to note that the welfare related decisions which farmers gave most importance to are those focused on reducing negative aspects of welfare (e.g., dominance, stress, health issues). Indeed, the central role farmers give to health and stress minimisation in their constructs of good animal welfare and what it entails, may in part explain their focus on health-related genetics decisions and their desire to support contentment in their animals. To see animals content was to indicate the absence of stress and, consequently, signal to farmers the presence of ‘good welfare’ (i.e., animals resource needs are met, and potential issues have been negated) and ‘good health’. Indeed, this ubiquitous desire amongst this study’s participants to encourage contentment is perhaps representative of a wider attitude amongst farmers; Spooner et al. [41] also find that pig farmers referred to ‘happy’ animals when describing a desired condition they sought for their pigs; namely, looking healthy and alert. Thus, it could be argued that there are parallels between farmers’ animal welfare-related perspectives and the wider animal welfare literature. Namely, farmers demonstrate a somewhat intuitive awareness of factors relevant for positive welfare, but consider their primary role is to minimise the negative experiences in their animals’ lives. Indeed, most farmers appear to hold the view that if they deal with the negative factors impacting their animals, then positive factors will arise naturally, or indirectly, as a result. Therefore, it could be argued that farmers’ current practices somewhat overlap with recommendations in the positive welfare literature, but their conscious management decisions are largely based on a belief that welfare is primarily about the reduction of negative issues. 

Farmers’ perspectives thus appear to possess some similitudes to the traditional animal welfare literature, which focuses more on the reduction of negatives, and the positive welfare literature which promotes the experiences of positives [42]. Determining why such overlaps between science and farmers exist is beyond the scope of this paper, however, it is worthwhile to theorise what may underlie it. One reason for the overlaps between farmers’ perspectives and the positive welfare literature may be the animal-based nature of positive welfare indicators. Hubbard and Scott [5], for example, found that when farmers and scientists independently produced welfare assessment measures which focused on using the animal as the source of welfare information, they produced very similar criteria. Thus, it could be theorised that due to their regular interactions with and observations of farm animals, both farmers and scientists form a common understanding of their needs and behaviours and thus consider similar factors as indicators of an animal’s welfare state. 

Nonetheless, it is important to recognise the limitations of this study and consider the potential for variance or misconceptions in farmers’ interpretations of some welfare indicators. For example, behavioural indicators of contentment can easily be confused with apathy and boredom [43]. In addition, the participant sample, although rich in insights and demonstrating a variety of different farming systems (e.g., zero-grazing dairy, out-wintering beef etc.), was mostly made up of dairy and beef and sheep farmers, with a small sample of pig and poultry farmers. Consequently, the sample is primarily composed of participants with an extensive system and a smaller number with an intensive system. As such, further interpretation of and reference to the findings of this study must be cognisant of the context in which they arise and how this affects, and potentially predisposes, positive welfare. For example, beef and sheep farmers, and some dairy farmers (e.g., pasture-based), may have greater opportunities to witness their animals engaging in happy-energised behaviours (e.g., play) because factors which trigger this (e.g., contrast from moving field) are an intrinsic part of their farming system. Similarly, pig and poultry producers may be more likely to use enrichment objects because there are legislative guidelines regarding their provision in these sectors (e.g., [44]). As such, it is important to be cautious when applying the findings of this data; a distinction must be made between farms with extensive and intensive systems, as they may not provide equivalent opportunities for positive welfare. Arguably, a more intensive system may require more direct management interventions to promote positive welfare than an extensive system; further research is needed to better understand the potential distinctions and overlaps between the two in this regard. Nevertheless, beyond these limitations, the insights of this study do highlight several areas which may help support the furtherance and acceptance of positive welfare indicators in livestock farming. 

## 6. Implications for the Development of Positive Welfare Indicators

Findings highlight several key points which may be beneficial to consider when developing positive welfare indicators. 

### 6.1. Farmers’ Value System

Previous research has shown that farmers’ values can influence their motivation to work with different aspects of animal welfare [45]. This was certainly evident in this study, as farmers who valued a more loosely structured farming system appeared to value animal autonomy more highly, and therefore were more motivated to provide this than those who valued a more highly structured farming system. This has implications for developing and communicating positive welfare indicators, as getting farmer buy-in for their implementation arguably requires indicators to fit with their value orientations. Indeed, the low uptake (10% of holdings in 2011) of the Scottish Government’s Animal Welfare Management Programme was thought to be caused, in part, by a lack of fit between individual variations in farming practice and aspects of the scheme [46]. Therefore, developing indicators which can flexibly translate to different farming systems, and thus be communicated in a manner which matches farmers’ values and fits with their preferred farming system, may enhance their motivation to employ them and the quality of their implementation. 

### 6.2. The Human-Animal Relationship

An interesting aspect of the human-animal relationship was how farmers’ discussions of experiencing personal positive affect (e.g., pride, joy, happiness, pleasure) occurred in the context of their animals experiencing positive affect (e.g., playing, looking content). This indicates the potential for animals experiencing greater opportunities for positive welfare to positively impact the human caretakers. The human-animal relationship literature tends to focus on the impact (often negative) that human caretakers have on farm animals [47,48]. However, there is growing acknowledgment that the human-animal relationship can also affect the on-farm experiences and job satisfaction of the human caretakers [49]. Set within a wider societal context of growing concerns over farmer mental health and well-being in the UK [50,51] and its impact on animal welfare [52], a potentially interesting way to develop and encourage acceptance of positive welfare indicators may be to strengthen awareness of the benefits for the whole farm; the human and the non-human animals. This has previously been noted by Yeates and Main [53], who argued that “where there is a sympathetic human-animal bond, when the human values the good things in an animal’s life, enriching the animal’s welfare can also enrich the carer’s welfare” (p. 294). Thus, emphasising this connection in the development and communication of positive welfare indicators may prove beneficial. 

### 6.3. Indicators Which Signal Farmers are Doing a ‘Good Job’

Farmers’ discussions of animal welfare, as illustrated in this study, often centre on what they provide and do for their animals, rather than what their animals are experiencing. Consequently, they seek indicators which signal to them that they are doing a good job (i.e., that they are providing what their animals need to thrive and be content). Indeed, although farmers provide a variety of resource, management and animal-based welfare inputs, ‘the doing’ or provision of good welfare to their animals is what indicates positive welfare to them. Thus, farmers value indicators which inform them of the efficacy of their management practices and inputs. Many of the positive welfare opportunities mentioned throughout could potentially have this role. For instance, play indicates to farmers that their animals’ resource or social interaction needs are supported, while contentment indicates they have fulfilled their duty of care. When developing welfare indicators, it is thus worth keeping such findings in mind, as they reveal what farmers want from a welfare indicator and how they wish to use them. To encourage farmer buy-in, it would arguably be of benefit to communicate what information positive welfare indicators could provide to farmers on an animal’s welfare state. One particularly important means to do this is to highlight potential links with productivity, as discussed further below.

### 6.4. Positive Welfare’s Connection with Productivity

It is well recognised that farmers almost ubiquitously use the productivity or performance of their animals as an indicator of their welfare state [41,54]. The primary perception of farmers, as discussed in this study, is that animals will not produce if they are not well cared for or not ‘happy’. Thus, the motivation of farmers to pursue welfare provisions which support animal productivity could potentially be harnessed to enhance the development and implementation of positive welfare indicators. Research is developing in this regard and studies have found that positive welfare opportunities, such as providing self-grooming brushes to dairy cattle [23] and positive social interactions in pigs [55], can contribute to animal productivity, along with the potential health benefits of positive welfare [42]. Thus, it may be relevant that such links are highlighted in the development of positive welfare indicators and particularly when communicating potential indicators to farmers.

## 7. Conclusions

This study explored the perspectives of livestock farmers on positive animal welfare to reveal insights relevant to the ongoing development of positive welfare indicators. It finds several positive welfare opportunities are freely elicited by farmers during their discussions of their management practices and their approaches to animal welfare. Namely, some provision for animal autonomy, an awareness of positive affect and a direct desire to promote contentment, a positive human-animal relationship, some support of positive social interactions, the use of genetics to improve health and, in some cases, the provision of environmental enrichments. Such factors fit well with current research on how to provide animals with greater opportunities for positive experiences. However, farmers possess a much more fluid and holistic approach to animal welfare—one that considers the specifics of their animals’ current situation, the characteristics of their individual farm, the wider economic environment and one that is underpinned by their personal values and preferences. This presents significant challenges for the development of positive welfare indicators, as there is a need for these to be flexible enough to adapt to the individual characteristics of different farmers and farms. Indeed, as this paper has argued throughout, encouraging the acceptance and implementation of positive welfare at the farm level requires the development of indicators which connect and resonate with farmers’ current norms, values and practices. As this study has revealed, there is potential for this by building on existing positive welfare indicators and communicating them in a manner which connects with farmers’ value systems, emphasises the whole farm (i.e., human and animal) benefits of a positive human-animal relationship, makes use of indicators which farmers can use to assess the efficacy of their management inputs and highlights connections between positive welfare and productivity. 

## Appendix A

**Table A1:** Format of narrative interviews with example of follow-up prompt questions.

Narrative-Inducing Question	Main Narration	Questioning Phase (Examples of Follow-up Prompts)
1. Can you tell me about what, in your experience, is a good life for a farm animal?	Active listening—noting further prompting questions	You mentioned you want them to be ‘happy and content’; how would you know that your animals are happy and content? When you say ‘keeping them properly’ can you give me some examples of what this involves? When you say, they are able to ‘play with their mates’ can you tell me how you manage this? You mentioned ‘giving the cow her choice’, can you describe to me how you would do this? You said that you wanted your animals to be outdoors; from an animal’s point of view, what makes this a good life?
2. Positive animal welfare—can you tell me about what comes to mind when you hear that?		Can you describe what you mean when you say going above and beyond; what does that involve? You describe it as going beyond just the basics; can you give me some examples of what these may be? You mentioned ‘positive environment’; can you give some examples of what you mean by this? When you say ‘something which is good and kind to the animal’ can you give some examples of what being good and kind involves?
3. Can you describe what motivates you to farm the way you do?		You mentioned seeing things in the past that you didn’t like, can you describe to me how this influences how you do things now? So, what is it about working directly with the animals that you find enjoyable? Could you perhaps describe some of the specific steps you took in that process, so how you designed your way of farming?

**Table A2:** Farmers’ demographic information.

Sector	Gender	Age	Farm Size (Ha)	Number of Animals	System
**Dairy**					
1	Male	30–40	130	100–200	Pasture
2	Male	50–60	137	200–300	Pasture
3	Male	18–30	62	100–200	Pasture
4	Male	30–40	343	700–800	Zero-grazed
5	Male	50–60	283	100–200	Pasture
6	Male	30–40	160	300–400	Pasture and robotic milking
7	Male	40–50	344	300–400	Pasture and zero-grazed, non-robotic and robotic milking
8	Male	30–40	100	100–200	Zero-grazed
9	Female	18–30	307	300–400	Zero-grazed
10	Male	40–50	776	1000–1500	Outdoor 365 days/year
11	Female	30–40	687	400–500	Pasture and zero-grazed
12	Male	40–50	176	100–200	Organic and robotic milking
13	Male	40–50	283	800–1000	Zero-grazed
**Beef and Sheep**					
1	Male	60–70	178	600–700	Indoor-wintered
2	Male	40–50	438	200–300	Indoor-wintered
3	Male	30–40	95	200–300	Outdoor-wintered
4	Male	50–60	230	400–500	Outdoor-wintered
5	Female	30–40	4	<100	Indoor-wintered
6	Male	50–60	100	400–500	Outdoor-wintered
7	Male	40–50	1011	200–300	Indoor-wintered
8	Male	60–70	60	400–500	Outdoor-wintered
9	Male	40–50	500	1000–1500	Outdoor-wintered & Indoor-wintered
10	Prefer not to say	40–50	750	1000–1500	Indoor-wintered
**Poultry (laying)**					
1	Male	30–40	141	10,000–15,000	Free range and organic
2	Male	50–60	95	120,000–130,000	Free range
**Mixed**					
1	Male	40–50	54	200–300	Free range (pig and poultry), organic (all species), indoor-wintered (beef), outdoor-wintered (sheep)
2	Male	30–40	230	1000–1500	Free range (poultry), straw-housed (pig), outdoor-wintered (sheep)
**Pig**					
1	Male	30–40	555	2000–3000	Housed (slats and straw)
